# Editorial Comment: Open Versus Laparoscopic Gubernaculum-Sparing Second-Stage Fowler-Stephens Orchiopexy for Intra-Abdominal Testis: A Long-Term Study

**DOI:** 10.1590/S1677-5538.IBJU.2022.05.03

**Published:** 2022-08-03

**Authors:** Luciano A. Favorito

**Affiliations:** 1 Universidade do Estado do Rio de Janeiro Rio de Janeiro RJ Brasil Unidade de Pesquisa Urogenital - Universidade do Estado do Rio de Janeiro - Uerj, Rio de Janeiro, RJ, Brasil

Guanglun Zhou ^1^, Jinjun Chen ^1^, Jianchun Yin ^1^, Xiaodong Liu ^1^, Jiahong Su ^1^, Shoulin Li ^1^

J Laparoendosc Adv Surg Tech A. 2022 Apr 18; Online ahead of print.

## COMMENT

The high abdominal undescended testis is a challenging situation in pediatric urology. The surgery to put the testicle in the scrotal region without harming its vascularization is very important for the treatment of these cases. Fowler-Stephens surgery performed in two times is the best strategy to preservation of vascular supply to the testis. In this important paper the authors shows the benefits of performing open versus laparoscopic gubernaculum-sparing second-stage Fowler-Stephens orchiopexy (FSO). The gubernaculum is the most important structure in the inguinoscrotal stage of testicular migration (1, 2). The knowledge of testicular and gubernaculum vascular anatomy is a key point in this procedure (3). In this paper the authors retrospectively studied a cohort of patients who underwent laparoscopic first-stage FSO and open versus laparoscopic gubernaculum-sparing second-stage FSO and concluded that the second-stage gubernaculum-sparing FSO achieved high testicular survival rates and satisfactory testicular positions. Neither the open nor laparoscopic approach appeared superior, because the overall testicular survival rates and incidence of testicular ascent and other complications were equivalent between both groups.
